# Pineal gland transcriptomic profiling reveals the differential regulation of lncRNA and mRNA related to prolificacy in STH sheep with two *FecB* genotypes

**DOI:** 10.1186/s12863-020-00957-w

**Published:** 2021-02-18

**Authors:** Chunyan Li, Xiaoyun He, Zijun Zhang, Chunhuan Ren, Mingxing Chu

**Affiliations:** 1 Key Laboratory of Animal Genetics, Breeding and Reproduction of Ministry of Agriculture and Rural Affairs, Institute of Animal Science, Chinese Academy of Agricultural Sciences, Beijing, 100193 China; 2 College of Animal Science and Technology, Anhui Agricultural University, Hefei, 230036 China

**Keywords:** LncRNAs, RNA-Seq, Pineal gland, Prolificacy, Sheep

## Abstract

**Background:**

Long noncoding RNA (lncRNA) has been identified as important regulator in hypothalamic-pituitary-ovarian axis associated with sheep prolificacy. However, little is known of their expression pattern and potential roles in the pineal gland of sheep. Herein, RNA-Seq was used to detect transcriptome expression pattern in pineal gland between follicular phase (FP) and luteal phase (LP) in *FecB*^*BB*^ (MM) and *FecB*^++^ (ww) STH sheep, respectively, and differentially expressed (DE) lncRNAs and mRNAs associated with reproduction were identified.

**Results:**

Overall, 135 DE lncRNAs and 1360 DE mRNAs in pineal gland between MM and ww sheep were screened. Wherein, 39 DE lncRNAs and 764 DE mRNAs were identified (FP vs LP) in MM sheep, 96 DE lncRNAs and 596 DE mRNAs were identified (FP vs LP) in ww sheep. Moreover, GO and KEGG enrichment analysis indicated that the targets of DE lncRNAs and DE mRNAs were annotated to multiple biological processes such as phototransduction, circadian rhythm, melanogenesis, GSH metabolism and steroid biosynthesis, which directly or indirectly participate in hormone activities to affect sheep reproductive performance. Additionally, co-expression of lncRNAs-mRNAs and the network construction were performed based on correlation analysis, DE lncRNAs can modulate target genes involved in related pathways to affect sheep fecundity. Specifically, *XLOC_466330*, *XLOC_532771*, *XLOC_028449* targeting *RRM2B* and *GSTK1*, *XLOC_391199* targeting *STMN1*, *XLOC_503926* targeting *RAG2*, *XLOC_187711* targeting *DLG4* were included.

**Conclusion:**

All of these differential lncRNAs and mRNAs expression profiles in pineal gland provide a novel resource for elucidating regulatory mechanism underlying STH sheep prolificacy.

**Supplementary Information:**

The online version contains supplementary material available at 10.1186/s12863-020-00957-w.

## Background

Reproduction, one of the major factors significantly affecting profitability of sheep production, is a complicated physiological process and determined by the integrated hypothalamic-pituitary-ovarian axis in breeding season [1]. Reproductive traits like litter size directly determine benefit of sheep production, are controlled by poly-gene at the micro level. How to undertake at molecular level to improve reproduction, thereby serving macro production is a hotspot in recent years. *BMPRIB*, *BMP15* [2] and *GDF9* [3] are major fecundity genes which significantly influence sheep prolificacy. *FecB* is a mutation in *BMPRIB* occurring in base 746 from A to G, one copy of this mutation significantly increases ovulation rate in sheep about 1.5 and two copies by 3.0 [4]. To date, this mutation has been detected in diverse sheep species such as Booroola Merino sheep (Australia) [5], Small Tail Han (STH) and Hu sheep (China) [6]. Wherein STH sheep is a famous native breed with year-round estrus and high fecundity, being officially recognized as one of the polytocous breeds in China. The average litter size and lambing rate of STH sheep are 2.61, 286.5%, respectively [7]. There are three genotypes based on effects of *FecB* mutation in STH sheep, namely *FecB*^*BB*^ (with two-copy *FecB* mutation), *FecB*^*B*+^ (with one-copy *FecB* mutation) and *FecB*^++^ (with no *FecB* mutation), which is closely correlated with litter size of ewes [8]. Therefore, this breed can be used as a classic model for study molecular mechanism of *FecB* gene regulation of reproductive traits in sheep.

Long noncoding RNA (lncRNA) is polymerase II transcript with length longer than 200 nucleotides that lacks the protein coding ability, its expression has high tissue specificity and distributes in cytoplasm or nucleus [9]. LncRNA is proposed to be the largest transcript class in mammalian transcriptome [10], less than 2% of mammalian genome actually code for protein, 70–90% is transcribed in some context as lncRNA, originally thought to be ‘transcriptional noise’ in genome. Subsequently, studies have gradually shown that lncRNA exerts important roles in various biological processes such as cell proliferation, apoptosis and differentiation [11], signal transduction [12], immune regulation [13]. In terms of reproduction, there have many reports on lncRNA. For example, Miao et al. (2017) compared transcripts in ovaries of low fecundity ewes and high fecundity ewes, found that differentially expressed (DE) lncRNA significantly enriched in the oxytocin signaling pathway [14]. Then, Feng et al. (2018) identified 5 lncRNAs and 76 mRNAs in ovaries of Hu sheep with high and low prolificacy, respectively [15]. Yang et al. (2020) analyzed lncRNA and mRNA in male sheep pituitary and found that 5 candidate lncRNAs and their targeted genes enriched in growth and reproduction related pathways [16]. Su et al. (2020) screened differential lncRNA through high-throughput sequencing, concluded that *XLOC-2222497* and its target *AKR1C1* could interact with progesterone in porcine endometrium for controlling pregnancy maintenance [17]. These studies indicated the presence and role of lncRNA in reproductive tissues. It is known that the sheep pineal gland as an important reproductive-related gland, that is closely related to hormone and signal transduction. However, studies on function of sheep lncRNA in this organ are limited.

In light of this, the study presented herein was focused on analyzing transcriptomics of pineal gland in STH sheep with *FecB*^*BB*^ (MM) and *FecB*^++^ (ww) genotypes, to determine the DE lncRNAs and genes, and predict their potential function that related to reproduction. Which is essential for better understanding the molecular mechanisms by lncRNAs regulate sheep reproduction with different genotypes, also providing insight for other female mammals.

## Results

### Summary of raw sequence reads

After removing low-quality sequences, a total of 288,342,450, 250,073,062, 289,224,844 and 277,834,922 clean reads with greater than 91.91% of Q30 were obtained in MM_F, MM_L, ww_F and ww_L, respectively. Approximately 86.10 to 92.89% of the reads were successfully mapped to the *Ovis aries* reference genome (Table 1).

**Table 1:** Summary of raw reads after quality control and mapping to the reference genome

Sample name	Raw reads number	Clean reads number	Clean reads rate (%)	Mapped reads	Mapping rate (%)	Q30 (%)
MM_F_P_1	99,577,992	96,579,902	96.99	89,494,204	92.66	95.25
MM_F_P_2	98,042,002	95,083,618	96.98	88,326,891	92.89	95.38
MM_F_P_3	99,359,596	96,678,930	97.30	89,144,759	92.21	93.97
MM_L_P_1	94,117,268	90,374,994	96.02	80,877,361	89.49	91.91
MM_L_P_2	84,813,806	81,105,250	95.63	69,833,669	86.10	92.63
MM_L_P_3	81,967,646	78,592,818	95.88	71,911,716	91.50	93.18
ww_F_P_1	90,655,762	88,791,808	97.94	81,552,108	91.85	94.37
ww_F_P_2	98,121,998	95,381,100	97.21	85,822,614	89.98	94.40
ww_F_P_3	108,614,426	105,051,936	96.72	94,957,100	90.39	93.18
ww_L_P_1	99,462,864	95,491,444	96.01	87,266,138	91.39	93.35
ww_L_P_2	85,154,530	83,228,220	97.74	75,517,349	90.74	93.11
ww_L_P_3	102,394,760	99,115,258	96.80	90,525,511	91.33	93.19

### Differential expression analysis of lncRNAs and mRNAs

**Fig. 1:**
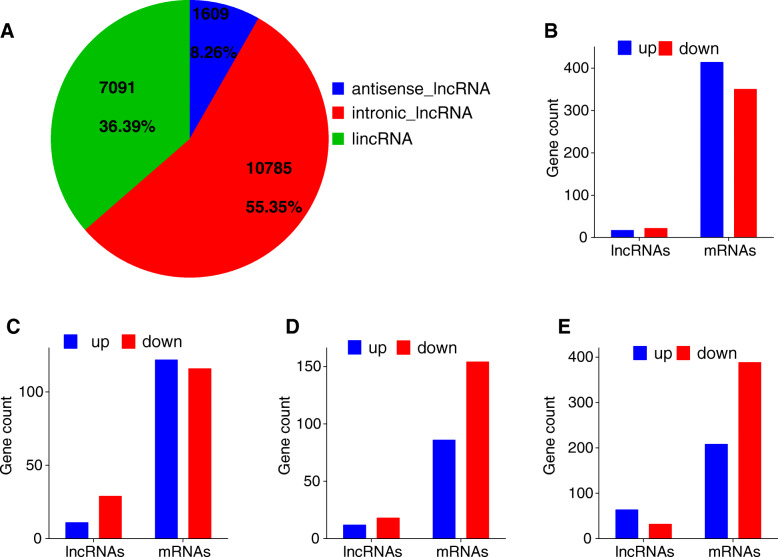
Gene expression characterization. **a** The classification and proportion of novel lncRNAs. **b** Histogram representing the numbers of upregulated and downregulated lncRNAs and mRNAs in sheep pineal body between MM_F_P and MM_L_P. **c** Histogram representing the numbers of upregulated and downregulated lncRNAs and mRNAs in sheep pineal body between MM_F_P and ww_F_P. **d** Istogram representing the numbers of upregulated and downregulated lncRNAs and mRNAs in sheep pineal body between MM_L_P and ww_L_P. **e** Histogram representing the numbers of upregulated and downregulated lncRNAs and mRNAs in sheep pineal body between ww_F_P and ww_L_P

A total of 21,282 lncRNAs (including 1797 known lncRNAs and 19,485 novel lncRNAs) and 43,674 mRNAs were identified from four groups (MM_F, MM_L, ww_F and ww_L) (Supplementary material 1A, B, 2). Overall, 10,785 intronic lncRNAs, 7091 intergenic lncRNAs (lincRNAs) and 1609 antisense lncRNAs were screened in the novel lncRNAs (Fig. 1a). Four comparison groups were set based on their genotypes and estrous cycle, MM_FP vs MM_LP, MM_FP vs ww_FP, MM_LP vs ww_LP, and ww_FP vs ww_LP. For MM_FP vs MM_LP, 17 lncRNAs and 414 mRNAs were upregulated, 22 lncRNAs and 350 mRNAs were downregulated (Fig. 1b, Supplementary material 3A, 4A). For MM_FP vs ww_FP, 11 lncRNAs and 122 mRNAs were upregulated, 29 lncRNAs and 116 mRNAs were downregulated (Fig. 1c, Supplementary material 3B, 4B). For MM_LP vs ww_LP, 12 lncRNAs and 86 mRNAs were upregulated, 18 lncRNAs and 154 mRNAs were downregulated (Fig. 1d, Supplementary material 3C, 4C). For ww_FP vs ww_LP, 64 lncRNAs and 208 mRNAs were upregulated, 32 lncRNAs and 388 mRNAs were downregulated (Fig. 1e, Supplementary material 3D, 4D). All DE lncRNAs (*P* < 0.05) and mRNAs (*P* < 0.05) were statistically significant.

**Fig. 2:**
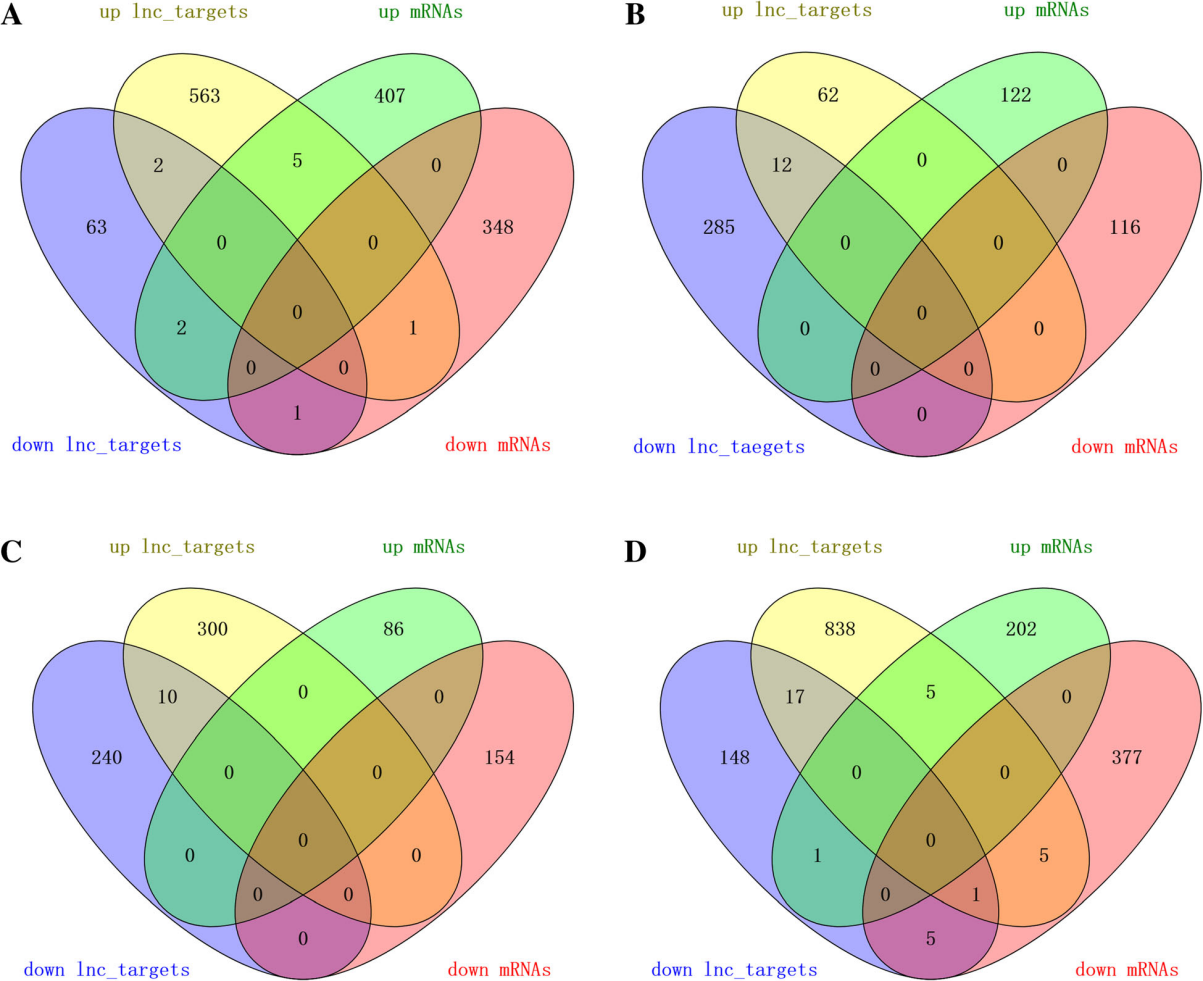
Venn diagram visualization of DE lncRNA_targets and mRNAs among four comparison groups. **a** Venn diagram representing the overlapping numbers of differentially expressed lncRNA_targets and mRNAs in MM_F_P vs MM_L_P. **b** Venn diagram representing the overlapping numbers of differentially expressed lncRNA_targets and mRNAs in MM_F_P vs ww_F_P. **c** Venn diagram representing the overlapping numbers of differentially expressed lncRNA_targets and mRNAs in MM_L_P vs ww_L_P. **d** Venn diagram representing the overlapping numbers of differentially expressed lncRNA_targets and mRNAs in ww_F_P vs ww_L_P

Venn diagram visually showed the numbers of common and unique DE lncRNA_targets and mRNAs among four comparison groups, as shown in Fig. 2a-d. In addition, distribution of these DE lncRNAs and mRNAs on chromosomes showed they were located on Chr2 (NC_019459.2), Chr3 (NC_019460.2), Chr1 (NC_019458.2) with greater proportion (Figures S1, S2, S3, S4, S5, S6, S7, S8), and reliable for their exon size and ORF length mostly within 1000 bp (Figure S9).

### GO analysis of the biological function of DE lncRNAs and mRNAs

**Fig. 3:**
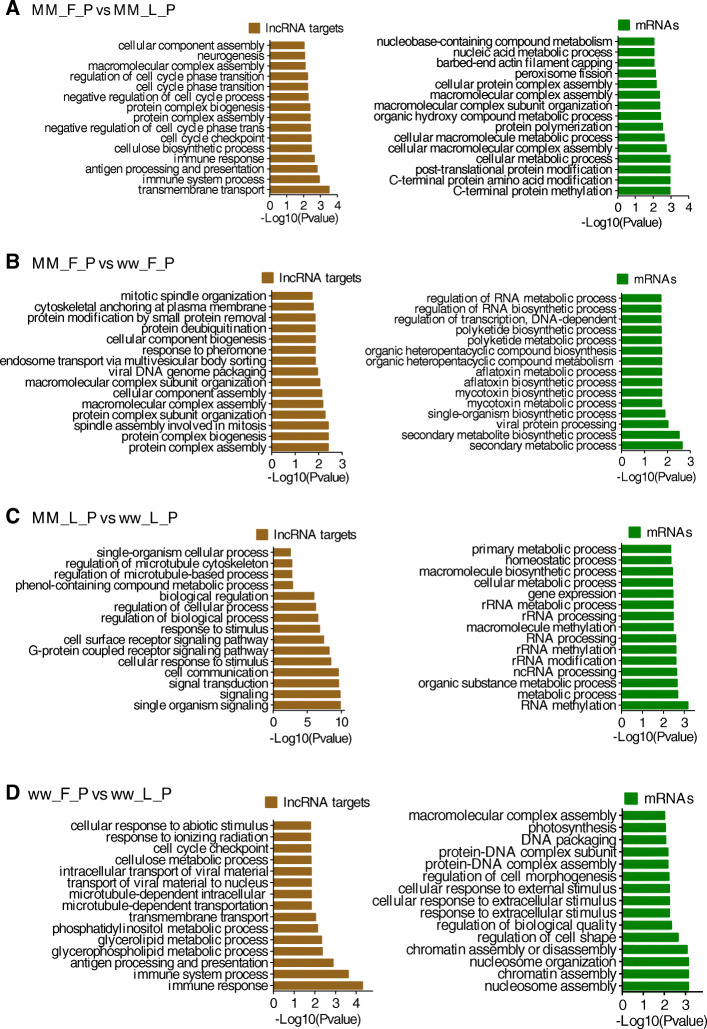
GO analyses of differentially expressed lncRNA targets and mRNAs. **a** The top 15 enrichment biological processes for differentially expressed lncRNA targets and mRNAs in MM_F_P vs MM_L_P. **b** The top 15 enrichment biological processes for differentially expressed lncRNA targets and mRNAs in MM_F_P vs ww_F_P. **c** The top 15 enrichment biological processes for differentially expressed lncRNA targets and mRNAs in MM_L_P vs ww_L_P. **d** The top 15 enrichment biological processes for differentially expressed lncRNA targets and mRNAs in ww_F_P vs ww_L_P

GO annotation enrichment was used to describe functions of the DE lncRNAs and mRNAs involved in cellular components, molecular function and biological processes, as shown in Fig. 3. Between MM_FP and MM_LP, targeted genes for DE lncRNAs were most enriched, and the terms were related to regulation of trans-membrane transport, antigen processing and presentation, immune system process. DE mRNAs were most enriched, the meaningful terms were related to the regulation of C-terminal protein methylation, C-terminal protein amino acid modification, post-translation protein modification, cellular macromolecular complex assembly and cellular metabolic process (Fig. 3a, Supplementary material 5A, 6A).

Between MM_FP and ww_FP, targeted genes for DE lncRNAs were enriched, the terms were related to regulation of protein complex assembly and biogenesis, protein complex subunit organization, spindle assembly involved in mitosis process. DE mRNAs were most enriched, the meaningful terms were related to regulation of secondary metabolic and biosynthetic process, viral protein processing, single-organism biosynthetic process (Fig. 3b, Supplementary material 5B, 6B).

Between MM_LP and ww_LP, targeted genes for DE lncRNAs were enriched, the terms were mainly related to regulation of single organism signaling, signal transduction, cellular response to stimulus and cellular communication. DE mRNAs were enriched, the meaningful terms were related to regulation of RNA methylation, metabolic process, organic substance metabolic process (Fig. 3c, Supplementary material 5C, 6C).

Between ww_FP and ww_LP, targeted genes for DE lncRNAs were enriched, the terms were related to regulation of immune response, glycerolipid metabolic process, cellular response to abiotic stimulus. DE mRNAs were enriched, the terms were related to regulation of nucleosome and chromatin assembly, nucleosome organization process (Fig. 3d, Supplementary material 5D, 6D).

### KEGG pathway analysis

**Fig. 4:**
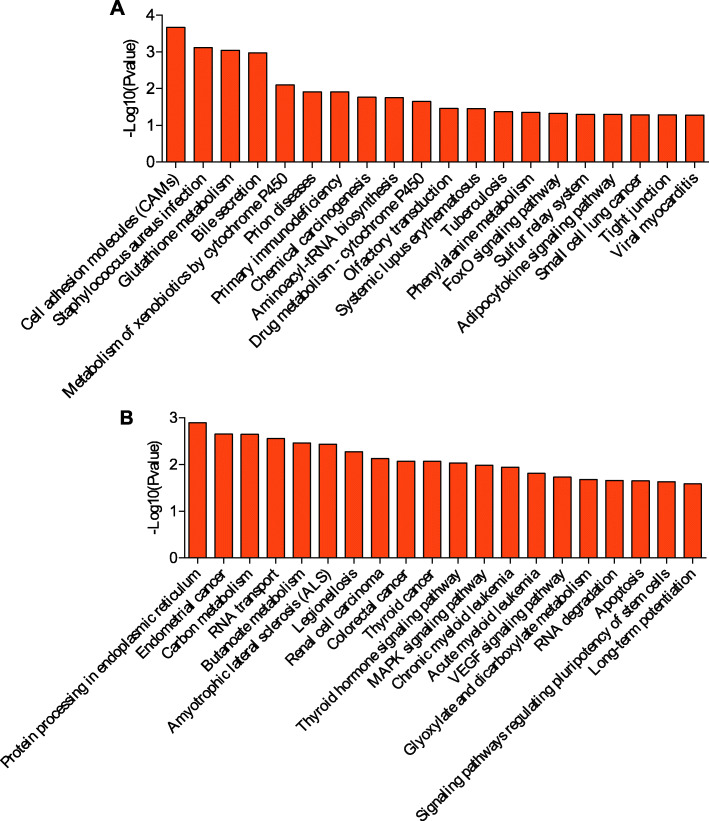
KEGG analyses of differentially expressed genes between MM_F_P and MM_L_P groups. **a** The top 20 KEGG enrichment pathways for differentially expressed lncRNA targets between MM_F_P and MM_L_P groups. **b** The top 20 KEGG enrichment pathways for differentially expressed mRNAs between MM_F_P and MM_L_P groups

**Fig. 5:**
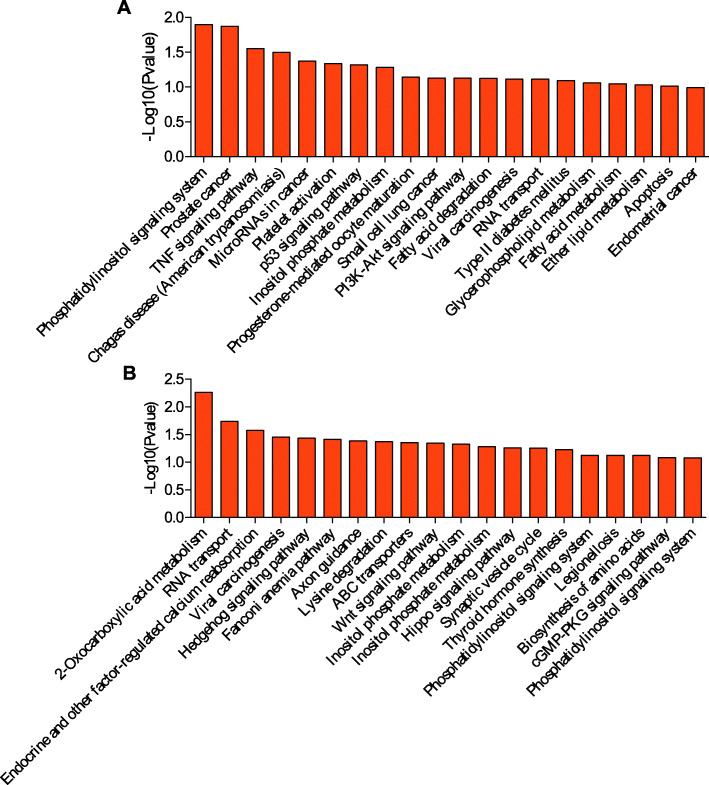
KEGG analyses of differentially expressed genes between MM_F_P and ww_F_P groups. **a** The top 20 KEGG enrichment pathways for differentially expressed lncRNA targets between MM_F_P and ww_F_P groups. **b** The top 20 KEGG enrichment pathways for differentially expressed mRNAs between MM_F_P and ww_F_P groups

**Fig. 6:**
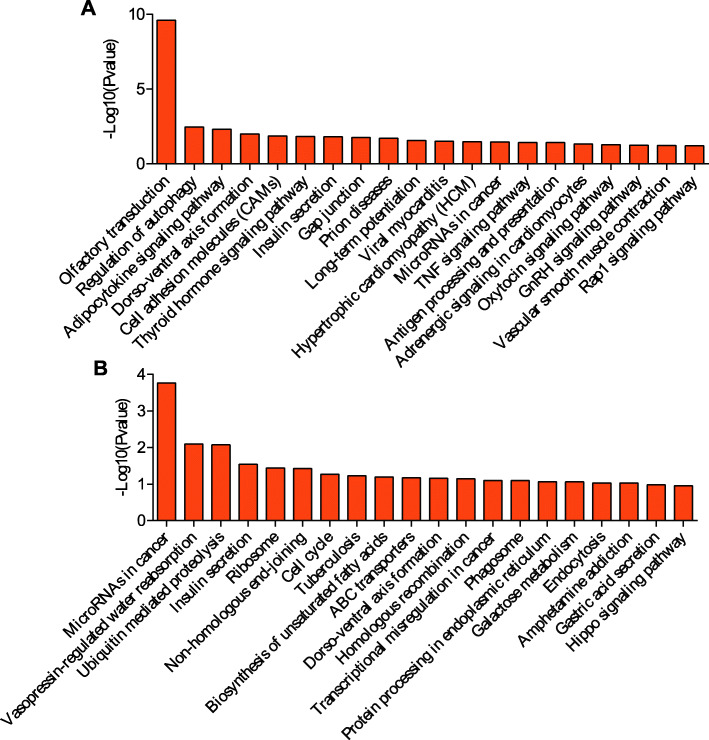
KEGG analyses of differentially expressed genes between MM_L_P and ww_L_P groups. **a** The top 20 KEGG enrichment pathways for differentially expressed lncRNA targets between MM_L_P and ww_L_P groups. **b** The top 20 KEGG enrichment pathways for differentially expressed mRNAs between MM_L_P and ww_L_P groups

**Fig. 7:**
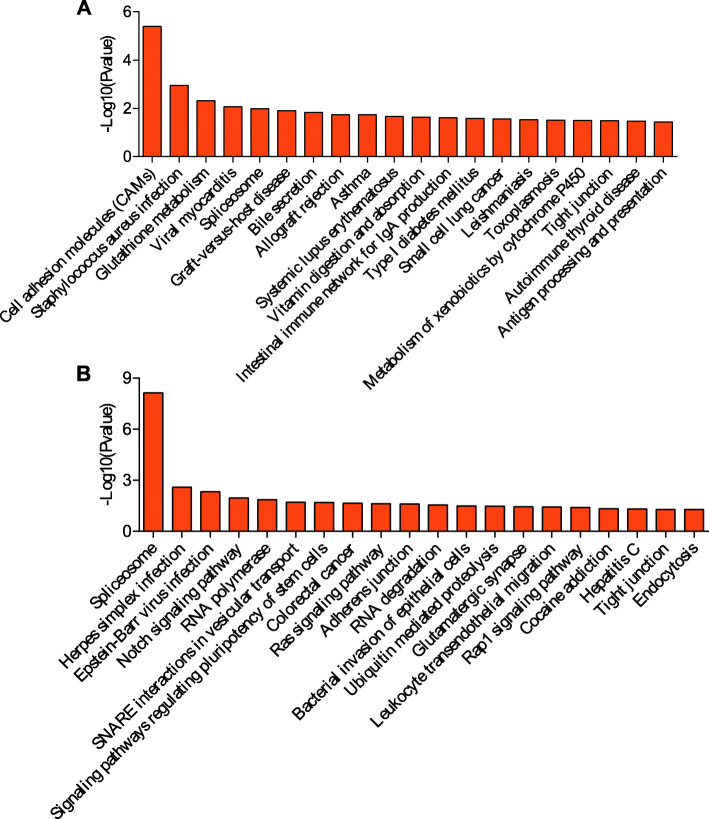
KEGG analyses of differentially expressed genes between ww_F_P and ww_L_P groups. **a** The top 20 KEGG enrichment pathways for differentially expressed lncRNA targets between ww_F_P and ww_L_P groups. **b** The top 20 KEGG enrichment pathways for differentially expressed mRNAs between ww_F_P and ww_L_P groups

KEGG is a primary public pathway database to understand potential function of DE genes. The top 20 pathways were showed in Figs. 4, 5, 6, 7. Between MM_FP and MM_LP, DE lncRNA targeted mRNAs were associated with pathways such as cell adhesion molecules (CAMs), glutathione (GSH) metabolism and bile secretion pathway (Fig. 4a, Supplementary material 7A). DE mRNAs were enriched in RNA transport, protein processing in endoplasmic reticulum and carbon metabolism pathway (Fig. 4b, Supplementary material 8A).

Between MM_FP and ww_FP, DE lncRNA targeted mRNAs were associated with pathways such as phosphatidylinositol signaling system, TNF signaling and p53 signaling pathway (Fig. 5a, Supplementary material 7B). With regard to DE mRNAs, which were enriched in 2-oxocarboxylic acid metabolism, RNA transport, endocrine and other factor-regulated calcium reabsorption pathways (Fig. 5b, Supplementary material 8B).

Between MM_LP and ww_LP, DE lncRNA targeted mRNAs were associated with pathways such as olfactory transduction, gap junction and thyroid hormone signaling pathway (Fig. 6a, Supplementary material 7C). With regard to DE mRNAs, which were enriched in ubiquitin mediated proteolysis, vasopressin-regulated water reabsorption, non-homologous end-joining and cell cycle (Fig. 6b, Supplementary material 8C).

Between ww_FP and ww_LP, DE lncRNA targeted mRNAs were associated with pathways such as cell adhesion molecules (CAMs), GSH metabolism and tight junction pathway (Fig. 7a, Supplementary material 7D). DE mRNAs were enriched in spliceosome, notch signal pathway, RNA polymerase and adherens junction, ras signaling pathway (Fig. 7b, Supplementary material 8D).

Hence, we acquired DE mRNAs closely related to reproductive signal pathways on the whole from above four comparison groups (Table S1).

### Interaction analysis of DE lncRNAs-mRNAs and function prediction

**Fig. 8:**
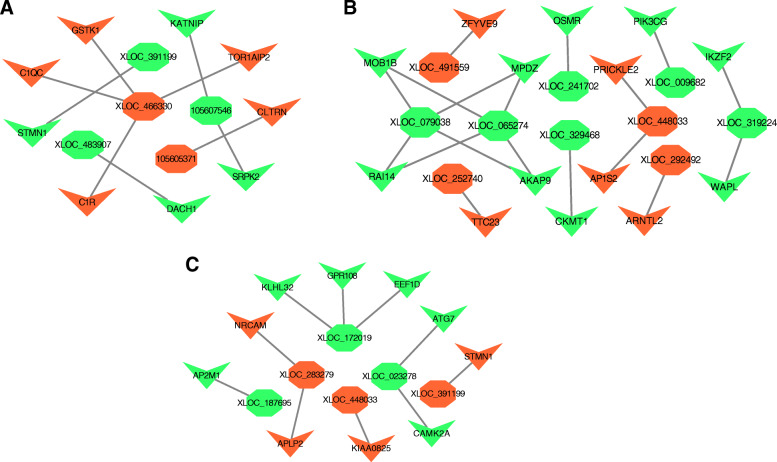
Construction of the DE lncRNAs-target mRNAs co-expression network. **a** Co-expression of DE lncRNA-mRNA after lncRNA targets coincided with DE mRNAs in MM_F_P vs MM_L_P. **b** Co-expression of DE lncRNA-mRNA after lncRNA targets coincided with DE mRNAs in MM_F_P vs ww_F_P. **c** Co-expression of DE lncRNA-mRNA after lncRNA targets coincided with DE mRNAs in MM_L_P vs ww_L_P. Tangerine and green represent upregulated and downregulated, respectively. Octagons and triangles represent lncRNAs and mRNAs, respectively

**Fig. 9:**
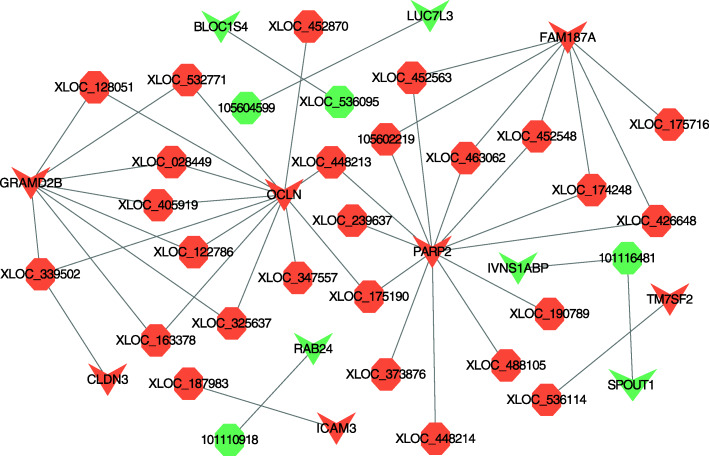
Co-expression of DE lncRNA-mRNA after lncRNA targets coincided with DE mRNAs in ww_F_P vs ww_L_P. Tangerine and green represent upregulated and downregulated, respectively. Octagons and triangles represent lncRNAs and mRNAs, respectively

To better understand the relationship between lncRNA and mRNA, we constructed network of co-expression of DE lncRNAs and DE target mRNAs, after screening the overlaps between target mRNAs and DE mRNAs in each comparison group, which indicated regulation of lncRNA and mRNA in reproduction (|Pearson correlation| >0.95). Between MM_FP and MM_LP, a total of 5 DE lncRNAs and 9 DE mRNAs were involved in the network, and it consists of 9 edges (Fig. 8a, Supplementary material 9A). Between MM_FP and ww_FP, a total of 10 DE lncRNAs and 14 DE mRNAs were involved in the network, and it consists of 18 edges (Fig. 8b, Supplementary material 9B). Between MM_LP and ww_LP, a total of 6 DE lncRNAs and 10 DE mRNAs were involved in the network, and it consists of 10 edges (Fig. 8c, Supplementary material 9C). Between ww_FP and ww_LP, a total of 30 DE lncRNAs and 12 DE mRNAs were involved in the network, and it consists of 47 edges (Fig. 9, Supplementary material 9D).

Based on analysis of co-expression, we screened DE lncRNAs and the DE target mRNAs that closely related to reproductive pathways in different reproductive cycles and genotypes sheep. In MM sheep, related pathways were enriched with 4 DE lncRNAs (*XLOC_466330*, *XLOC_391199*, *XLOC_503926*, *XLOC_517836*) and 4 DE targets (*RRM2B*, *GSTK1*, *STMN1*, *RAG2*) (Table 2). In ww sheep, related pathways were enriched with 6 DE lncRNAs (*XLOC_532771*, *XLOC_347557*, *XLOC_339502*, *XLOC_187711*, *XLOC_028449*, *105,604,037*) and 7 DE targets (*GPX2*, *LOC101111397*, *RRM2B*, *GPX1*, *GSTK1*, *MGST1*, *DLG4*) (Table 3). Additionally, related pathways were enriched by 7 DE lncRNAs (*XLOC_448033*, *XLOC_252740*, *XLOC_241702*, *XLOC_079038*, *XLOC_078000*, *XLOC_065274*, *XLOC_009682*) and 9 DE targets (*DCT*, *PLCB4*, *PIK3CG*, *S1PR1*, *BRCA1*, *OSMR*, *PDGFD*, *RRM2B*, *CHEK1*) in two groups of sheep (MM vs ww) at follicular phase (Table 4). And they were also enriched by 3 DE lncRNAs (*XLOC_283279*, *XLOC_187695*, *XLOC_023278*) and 11 DE targets (*PRKACB*, *PRKAA1*, *PPP2R2A*, *PLCB4*, *NOS3*, *NCOA2*, *MAP2K6*, *MAP2K1*, *LOC101121082*, *LOC101111988*, *CAMKK2*) in two groups of sheep (MM vs ww) at luteal phase (Table 5).

**Table 2:** Summary of co-expression of differential genes closely related to reproductive cycle (follicular phase vs luteal phase) in MM sheep

lncRNA_id	mRNA_id	mRNA_gene symbol	pathway_term	Pearson_correlation	***P***_value	regulation
XLOC_466330	101,123,639	RRM2B	Glutathione metabolism	0.975416236	6.78601E-08	up
i-	101,114,517	GSTK1	i-	0.9556985	1.24739E-06	i-
XLOC_391199	101,113,917	STMN1	MAPK signaling pathway	0.966221936	3.27202E-07	down
XLOC_503926	101,107,628	RAG2	FoxO signaling pathway	0.962879183	5.21528E-07	i-
XLOC_517836	i-		i-	0.960254429	7.30644E-07	i-

Note: “i-” represents the identical information with previous one in the same column

**Table 3:** Summary of co-expression of differential genes closely related to reproductive cycle (follicular phase vs luteal phase) in ww sheep

lncRNA_id	mRNA_id	mRNA_gene symbol	pathway_term	Pearson_correlation	***P***_value	regulation
XLOC_347557	101,110,596	GPX2	Glutathione metabolism	0.980030026	2.41896E-08	up
XLOC_532771	101,111,397	LOC101111397	i-	0.958822484	8.69982E-07	i-
i-	101,123,639	RRM2B	i-	0.964541292	4.15934E-07	i-
XLOC_339502	100,820,742	GPX1	i-	0.951981058	1.85453E-06	i-
XLOC_028449	101,114,517	GSTK1	i-	0.962757353	5.30034E-07	i-
105,604,037	101,103,462	MGST1	i-	0.966985892	2.92214E-07	down
XLOC_187711	101,116,743	DLG4	Hippo signaling pathway	0.963455638	4.82742E-07	i-

Note: “i-” represents the identical information with previous one in the same column

**Table 4:** Summary of co-expression of differential genes closely related to reproduction in different genotypes (MM vs ww) sheep at follicular phase

lncRNA_id	mRNA_id	mRNA_gene symbol	pathway_term	Pearson_correlation	***P***_value	regulation
XLOC_252740	100,170,232	DCT	Melanogenesis	0.953066536	1.65724E-06	up
XLOC_448033	101,106,864	PLCB4	Melanogenesis, Estrogen signaling pathway, Thyroid hormone signaling pathway	0.999712169	1.55499E-17	i-
XLOC_252740	101,102,896	PIK3CG	Estrogen signaling pathway, Thyroid hormone signaling pathway, AMPK signaling pathway, FoxO signaling pathway, Progesterone-mediated oocyte maturation, PI3K-Akt signaling pathway	0.993113969	1.20533E-10	i-
XLOC_009682	i-	i-	i-	0.963224057	4.98038E-07	i-
XLOC_448033	101,115,839	S1PR1	FoxO signaling pathway	0.959110126	8.40426E-07	i-
XLOC_078000	101,108,584	BRCA1	PI3K-Akt signaling pathway	0.950186768	2.22112E-06	down
XLOC_241702	101,105,948	OSMR	PI3K-Akt signaling pathway	0.952480425	1.76158E-06	i-
XLOC_065274	101,117,784	PDGFD	i-	0.98347647	9.43554E-09	i-
XLOC_079038	i-	i-	i-	0.965453824	3.65662E-07	i-
XLOC_078000	101,123,639	RRM2B	p53 signaling pathway	0.972189367	1.25045E-07	i-
XLOC_079038	i-	i-	i-	0.960693815	6.91655E-07	i-
XLOC_065274	i-	i-	i-	0.950086125	2.24327E-06	i-
i-	101,111,403	CHEK1	i-	0.950790069	2.09198E-06	i-

Note: “i-” represents the identical information with previous one in the same column

**Table 5:** Summary of co-expression of differential genes closely related to reproduction in different genotypes (MM vs ww) sheep at luteal phase

lncRNA_id	mRNA_id	mRNA_gene symbol	pathway_term	Pearson_correlation	***P***_value	regulation
XLOC_283279	101,123,635	MAP2K1	Thyroid hormone signaling pathway, GnRH signaling pathway, Progesterone-mediated oocyte maturation, Melanogenesis, Estrogen signaling pathway	0.977250667	4.61908E-08	up
i-	101,108,785	PRKACB	i-	0.975336415	6.89597E-08	i-
i-	i-	i-	i-	0.992013105	2.52552E-10	i-
i-	101,106,864	PLCB4	i-	0.977111161	4.76134E-08	i-
i-	101,104,249	NCOA2	Thyroid hormone signaling pathway	0.99755373	6.87071E-13	i-
i-	101,121,082	LOC101121082	i-	0.955392571	1.29039E-06	i-
i-	101,115,047	MAP2K6	GnRH signaling pathway	0.992233715	2.19628E-10	i-
i-	101,111,988	LOC101111988	Progesterone-mediated oocyte maturation, Melanogenesis	0.961681175	6.10058E-07	i-
i-	443,077	NOS3	Estrogen signaling pathway	0.954200987	1.46923E-06	i-
XLOC_187695	101,110,299	PPP2R2A	AMPK signaling pathway	0.997421407	8.93918E-13	down
i-	101,103,267	CAMKK2	AMPK signaling pathway, Oxytocin signaling pathway	0.965609431	3.57594E-07	i-
i-	101,103,425	PRKAA1	i-	0.997533947	7.15282E-13	i-
XLOC_023278	i-	i-	i-	0.956416823	1.15089E-06	i-
i-	443,453	CAMK2A	Oxytocin signaling pathway	0.966297838	3.23584E-07	i-

Note: “i-” represents the identical information with previous one in the same column

## Discussion

Studies have found that lncRNA is involved in multiple reproductive functions such as spermatogenesis [18], placentation [19], signaling pathway of sex hormone response [20, 21] and gonadgenesis [22]. It is known that the melatonin synthesized in pineal gland is closely related to the estrus cycle [23]. Herein, the study focused on examining expression profiles of pineal gland lncRNAs and mRNAs in sheep with two genotypes at different phases of estrous cycle using RNA-Seq technology. Analysis of relationship between DE lncRNAs and mRNAs by generating a co-expression network. To our knowledge, this is the first genome-wide analysis of pineal gland in sheep with different genotypes, and might provide valuable resource for searching functional lncRNAs associated with sheep prolificacy.

In present study, we screened 21,282 lncRNAs and 43,674 mRNAs. LncRNAs have synergetic relationship with mRNAs as most lncRNAs are located near protein-coding genes [24, 25]. Additionally, wide location of lncRNAs in chromosomes of sheep indicated its pluripotency. Obviously, distribution ratio of lncRNAs and mRNAs on Chr2 (NC_019459.2), Chr3 (NC_019460.2), Chr1 (NC_019458.2) were greater than those on other chromosomes, which could be explained by close relationship between three chromosomes and pineal gland function. The exon size and ORF length of lncRNAs and mRNAs are mostly within 1000 bp. These results showed the potential lncRNAs were reliable in the pineal gland.

Overall, we screened 135 (39 + 96) DE lncRNAs and 1360 (764 + 596) DE mRNAs in pineal gland at follicular and luteal phases between high and low prolificacy STH sheep (WW vs ww). GO annotation and KEGG enrichment analysis of top 20 terms indicated that DE mRNAs were enriched in reproduction-related pathways such as GnRH, cGMP-PKG, thyroid hormone, MAPK, phototransduction, circadian rhythm, steroid biosynthesis, hippo, mTOR and melanogenesis. It is well known that productive cycle of mammals is regulated through association or acting alone of hypothalamic-pituitary-thyroid (HPT) axis and hypothalamic-pituitary-gonadal (HPG) axis [26, 27]. In the HPT axis, thyrotropin-releasing hormone (TRH) produced in hypothalamus stimulates pituitary to secrete thyroid-stimulating hormone (TSH), which promotes TH synthesis in the thyroid gland [26, 28]. In the HPG axis, GnRH in hypothalamus regulates synthesis and secretion of FSH and LH in the anterior pituitary. These two hormones stimulate gonadal estrogen synthesis by binding to their receptors for affecting development and maturation of follicles and the ewes litter size. Estrogen in turn positively or negatively acts GnRH synthesis, and affects *FSHβ* gene expression, a hormone specific *β* subunit that is mainly regulated by GnRH [29, 30]. In the process, binding of GnRH to its receptor activates signaling cascades like MAPK, PI3K-Akt [31]. MAPK pathway is essential for cell proliferation and differentiation, survival, death and transformation [32, 33]. PI3K-Akt can interact with mTOR pathway to effectively regulate growth hormone in pituitary [34]. Additionally, pathways as hippo modulates organ size growth by controlling stem cell activity, proliferation and apoptosis. For instance, its’ effect on the development of pituitary progenitor cells [35]. Our results showed that DE genes like *AKT3*, *MYC*, *PIK3CB*, *MAP2K2*, *PLCB1* and *TEAD1* related to thyroid hormone, MAPK, cGMP-PKG, hippo, and up regulated, while *CTNNB1*, *YAP1*, *PIK3CG*, *TEAD1*, *CAMK2A*, *CACNA1D* mainly related to hippo, thyroid hormone, cGMP-PKG, AMPK, GnRH, oxytocin, circadian entrainment, and down regulated, which implied the important roles of these genes mainly involved in regulation of hormone-related pathways. It’s worth exploring their function in pineal gland as candidate genes.

Co-expression analysis of differential lncRNA-mRNA and functional prediction of target genes revealed that lncRNA affects sheep fecundity by modulating genes associated with above signaling pathways and biological processes. In *FecB*^*BB*^ genotype sheep, *XLOC_466330* and the targets (*RRM2B*, *GSTK1*) up regulated at follicular phase, which related to GSH metabolism. Whereas *XLOC_391199* and the target (*STMN1*), *XLOC_503926*, *XLOC_517836* and the target (*RAG2*) up regulated at luteal phase, which mainly enriched in MAPK, FoxO signaling pathways, respectively. In *FecB*^++^ genotype sheep, *XLOC_347557* and the target (*GPX2*), *XLOC_532771* and the targets (*LOC101111397*, *RRM2B*), *XLOC_339502* and the target (*GPX1*), *XLOC_028449* and the target (*GSTK1*) up regulated at follicular phase, which also related to GSH metabolism. Meanwhile, *105,604,037* and the target (*MGST1*), *XLOC_187711* and the target (*DLG4*) down regulated at the same phase that related to GSH metabolism and hippo signaling. Wherein *GSTK1* and *RRM2B* involved in GSH metabolism at follicular phase, but their targeted regulators lncRNAs were markedly different among two *FecB* genotypes. *RRM2B* gene encodes p53R2, and p53R2 is expressed at all phases of cell cycle to ensure ample supply of mitochondrial DNA [36]. *GSTK1* gene encodes a member of GSTK superfamily of enzymes that function in cellular mitochondria and peroxisomes detoxification during GSH metabolism [37, 38], a critical pathway protecting cells from free radicals and oxidative damage, could increase intracellular NADPH [39]. With increase of NADPH oxidase, ROS level tend to be low, whereas the level of intracellular ATP enhanced, as well as mitochondrial activity, which promote oocyte maturation [40], and so forth, the other DE genes involved in GSH metabolism were also novel direction of interest for their effects on the downstream reproductive system.

Furthermore, DE target genes like *STMN1* is a highly conserved gene that codes for cytoplasmic phosphoproteins, acting role in cell cycle progression, signal transduction and cell migration through diverse intracellular signaling pathways. Studies have found the potential role of STMN1 in regulation of hormone secretion in rodent pituitary and insulinoma cell lines [41]. Over-expression of STMN1 stimulates progesterone production by modulating the promoter activity of *Star* and *Cyp11a1* in mouse granulosa cells [42]. Besides, *RAG2* is indispensable for generation of antigen receptor diversity in immune cells [43]. We found *STMN1*, *RAG2* were down regulated at follicular phase in *FecB*^*BB*^ sheep, and mainly related to MAPK, FoxO signaling pathways, respectively. *DLG4* was down regulated at follicular phase in *FecB*^*++*^ sheep and enriched in hippo signaling term. As known that *DLG4* encodes a member of MAGUK family, is widely expressed and playing an essential role in regulation of cellular signal transduction, circadian entrainment [44]. Taken together, the DE lncRNAs identified in this study might cooperate with their target genes and DE genes to regulate pineal gland physiological function, and involved in hormone synthesis for effecting reproductive cycle and final lambing.

## Conclusion

In summary, the pineal gland transcriptomic study reveals differential regulation of lncRNAs and mRNAs related to prolificacy in sheep with different *FecB* genotyping. We screened several sets of target genes of DE lncRNAs and DE genes under reproductive cycle and genotypes, they were annotated to multiple biological processes such as phototransduction, circadian rhythm, melanogenesis, GSH metabolism and steroid biosynthesis, which directly or indirectly participate in hormone activities to affect sheep reproductive performance. Additionally, we predicted potential role of these DE lncRNAs and constructed network of lncRNAs-mRNAs to expand our understanding. All of these differential lncRNAs and mRNAs expression profiles provide a novel resource for elucidating regulatory mechanism underlying STH sheep prolificacy.

## Methods

### Ethics statement

Experimental animals in this study were authorized by the Science Research Department (in charge of animal welfare issues) of the Institute of Animal Science, Chinese Academy of Agricultural Sciences (IAS-CAAS; Beijing, China). Additionally, ethical approval of animal survival and the sample collection was given by the animal ethics committee of IAS-CAAS (No. IAS2019–49).

### Animals preparation

Animals were from a core population of STH sheep in Luxi district of Shandong province, China. We collected jugular vein blood of healthy non-pregnant sheep aged 2–4 years (*n* = 890), to identify the *FecB* genotypes using TaqMan probe [45]. Then, 12 sheep (6 MM and 6 ww, respectively) with no significant difference in age, weight, height, body length, chest circumference and tube circumference were selected for this experiment.

Twelve sheep were managed and raised on a farm, with free access to water and feed. All sheep were processed by estrus synchronization with Controlled Internal Drug Releasing device (CIDR, progesterone 300 mg, Inter Ag Co., Ltd., New Zealand) for 12 days. 3 MM and 3 ww ewes were euthanized (Intravenous pentobarbital at 100 mg/kg) on the 50th hour after CIDR removal, pineal tissues were collected (follicular phase, MM_FP and ww_FP, respectively). The other 6 sheep were euthanized (Intravenous pentobarbital at 100 mg/kg) on the 7th day after CIDR removal, and pineal tissues were collected (luteal phase, MM_LP and ww_LP, respectively) [21]. Obtained samples were stored immediately at − 80 °C for the next step.

### RNA extraction and detection

Total RNA was extracted from 12 samples using TRIzol reagent (Invitrogen, Carlsbad, CA, USA) according to manufacturer’s instruction. 1% agarose gel was used to monitor whether isolated RNA was degraded or contaminated. Quality, integrity and concentration of RNA were assessed by NanoPhotometer® spectrophotometer (IMPLEN, CA, USA), RNA Nano 6000 Assay Kit of the Bioanalyzer 2100 system (Agilent Technologies, CA, USA) and Qubit® RNA Assay Kit in Qubit® 2.0 Flurometer (Life Technologies, CA, USA), respectively. Among them, the ratio of intact RNA with RIN ≥ 7, 28S: 18S ≥ 1.5:1.

### Construction of RNA libraries and sequencing

A total amount of 3 μg RNA per sample was used as input material for the RNA sample preparation. Firstly, rRNA was removed by Epicentre Ribo-zero™ rRNA Removal Kit (Epicentre, USA) and rRNA free residue was cleaned up by ethanol precipitation. Subsequently, libraries were generated using the rRNA-depleted RNA by NEBNext® Ultra™ Directional RNA Library Prep Kit for Illumina® (NEB, USA) following manufacturer’s recommendation. After the clustering of the index-coded samples was performed on a cBot Cluster Generation System using TruSeq PE Cluster Kit v3-cBot-HS (Illumia), libraries were then sequenced through an Illumina Hiseq 4000 platform and 150 bp paired-end reads were generated.

### Reference genome mapping and transcriptome assembly

Raw reads of fast-q format were firstly processed through in-house perl scripts to obtain clean reads. At the same time, Q20, Q30 and GC content of the clean data were calculated. All downstream analyses were based on the high quality clean reads. HISAT2 v. 2.0.4 was used to align paired-end clean reads of each sample to sheep reference genome *Oar* v. 4.0 [46]. The mapped reads of each sample were assembled by StringTie v. 1.3.1 [46].

### Identification of potential lncRNA candidates

Potential lncRNA candidates were identified using the following workflow. (1) Transcripts with uncertain chain direction were removed by Cuffmerge. (2) Transcripts length > 200 nt with exon number ≥ 2 were selected. (3) Cuffcompare v. 2.1.1. was used to compare different classes of class_code annotated by “i”, “u” and “x” that were retained, which corresponded to intronic, intergenic, and anti-sense transcripts, respectively. (4) Transcripts with FPKM ≥0.5 were obtained after calculating the expression level of each transcript by Cuffdiff v. 2.1.1. (5) Three tools of CNCI v.2.0 [47], CPC v. 2.81 [48] and PFAM v.1.3 [49] were used to predict the protein-coding potential. After that, Pfam was implemented to exclude transcripts that overlapped with any protein-coding genes. Intersection of these transcripts without coding potential was used as the lncRNA data set. Additionally, mRNAs were obtained from the same RNA-seq libraries in this study.

### Analysis of differentially expressed genes

The fragments per kilobase of transcript per million reads mapped (FPKM) value was used to estimate the expression levels of transcripts (lncRNAs and mRNAs) [50]. For experiments with three biological replicates, the R package DEseq2 was used to identify differentially expressed transcripts after a negative binomial distribution [51]. LncRNAs and mRNAs with *P-*value < 0.05 and a fold change (FC) > 2.0 were considered as differentially expression between two groups of data.

### Bioinformatics analysis

LncRNA targets could be predicted by the correlation or co-expression of lncRNA and mRNA expression levels between samples. Among them, Pearson correlation coefficient (PCC) was used to analyze the correlation between lncRNA and mRNA among samples, mRNAs with |PCC| ≥0.95 for functional enrichment to predict lncRNAs [52]. Statistical enrichment of DE lncRNA targets and DE mRNAs in GO annotation and KEGG pathway were evaluated, *P*-value ≤0.05 defined as the significant threshold, significance of the term enrichment analysis was corrected by FDR and corrected *P*-value (*Q*-value) was obtained [53].

### Construction of co-expression networks

To predict function of DE lncRNAs and their target genes in sheep reproduction, a network based on lncRNAs and mRNAs was bulit using Cytoscape software (v. 3.8.0) [54].

### Statistical analysis

All data were assessed as the “means ± SD”. Student’s *t*-test was performed and *P* < 0.05 was considered statistically significant.

## Supplementary Information

**Additional file 1: Figure S1.** Distribution of DE lncRNAs on chromosomes in MM_FP vs MM_LP.

**Additional file 2: Figure S2.** Distribution of DE lncRNAs on chromosomes in MM_FP vs ww_FP.

**Additional file 3: Figure S3.** Distribution of DE lncRNAs on chromosomes in MM_LP vs ww_LP.

**Additional file 4: Figure S4.** Distribution of DE lncRNAs on chromosomes in ww_FP vs ww_LP.

**Additional file 5: Figure S5.** Distribution of DE mRNAs on chromosomes in MM_FP vs MM_LP.

**Additional file 6: Figure S6.** Distribution of DE mRNAs on chromosomes in MM_FP vs ww_FP.

**Additional file 7: Figure S7.** Distribution of DE mRNAs on chromosomes in MM_LP vs ww_LP.

**Additional file 8: Figure S8.** Distribution of DE mRNAs on chromosomes in ww_FP vs ww_LP.

**Additional file 9: Figure S9.** Density distribution of candidate transcripts.

**Additional file 10: Table S1.** Overview of DE mRNAs closely related to reproductive signal pathways.

**Additional file 11: Supplementary material 1A.** Total set of known lncRNAs were identified from four groups. **Supplementary material 1B.** Total set of novel lncRNAs were identified from four groups.

**Additional file 12: Supplementary material 2.** Total set of mRNAs were identified from four groups.

**Additional file 13: Supplementary material 3A.** Total set of lncRNAs were up- and down- regulated in MM_FP vs MM_LP. **Supplementary material 3B.** Total set of lncRNAs were up- and down- regulated in MM_FP vs ww_FP. **Supplementary material 3C.** Total set of lncRNAs were up- and down- regulated in MM_LP vs ww_LP. **Supplementary material 3D.** Total set of lncRNAs were up- and down- regulated in ww_FP vs ww_LP.

**Additional file 14: Supplementary material 4A.** Total set of mRNAs were up- and down- regulated in MM_FP vs MM_LP. **Supplementary material 4B.** Total set of mRNAs were up- and down- regulated in MM_FP vs ww_FP. **Supplementary material 4C.** Total set of mRNAs were up- and down- regulated in MM_LP vs ww_LP. **Supplementary material 4D.** Total set of mRNAs were up- and down- regulated in ww_FP vs ww_LP.

**Additional file 15: Supplementary material 5A.** GO enrichment of differentially expressed lncRNA targets in MM_FP vs MM_LP. **Supplementary material 5B.** GO enrichment of differentially expressed lncRNA targets in MM_FP vs ww_FP. **Supplementary material 5C.** GO enrichment of differentially expressed lncRNA targets in MM_LP vs ww_LP. **Supplementary material 5D.** GO enrichment of differentially expressed lncRNA targets in ww_FP vs ww_LP.

**Additional file 16: Supplementary material 6A.** GO enrichment of differentially expressed mRNAs in MM_FP vs MM_LP. **Supplementary material 6B.** GO enrichment of differentially expressed mRNAs in MM_FP vs ww_FP. **Supplementary material 6C.** GO enrichment of differentially expressed mRNAs in MM_LP vs ww_LP. **Supplementary material 6D.** GO enrichment of differentially expressed mRNAs in ww_FP vs ww_LP.

**Additional file 17: Supplementary material 7A.** Total set of the top 20 KEGG enrichment pathways for differentially expressed lncRNA targets in MM_FP vs MM_LP. **Supplementary material 7B.** Total set of the top 20 KEGG enrichment pathways for differentially expressed lncRNA targets in MM_FP vs ww_FP. **Supplementary material 7C.** Total set of the top 20 KEGG enrichment pathways for differentially expressed lncRNA targets in MM_LP vs ww_LP. **Supplementary material 7D.** Total set of the top 20 KEGG enrichment pathways for differentially expressed lncRNA targets in ww_FP vs ww_LP.

**Additional file 18: Supplementary material 8A.** Total set of the top 20 KEGG enrichment pathways for differentially expressed mRNAs in MM_FP vs MM_LP. **Supplementary material 8B.** Total set of the top 20 KEGG enrichment pathways for differentially expressed mRNAs in MM_FP vs ww_FP. **Supplementary material 8C.** Total set of the top 20 KEGG enrichment pathways for differentially expressed mRNAs in MM_LP vs ww_LP. **Supplementary material 8D.** Total set of the top 20 KEGG enrichment pathways for differentially expressed mRNAs in ww_FP vs ww_LP.

**Additional file 19: Supplementary material 9A.** Co-expression details of DE lncRNA-mRNA after lncRNA targets coincided with DE mRNAs in MM_FP vs MM_LP. **Supplementary material 9B.** Co-expression details of DE lncRNA-mRNA after lncRNA targets coincided with DE mRNAs in MM_FP vs ww_FP. **Supplementary material 9C.** Co-expression details of DE lncRNA-mRNA after lncRNA targets coincided with DE mRNAs in MM_LP vs ww_LP. **Supplementary material 9D.** Co-expression details of DE lncRNA-mRNA after lncRNA targets coincided with DE mRNAs in ww_FP vs ww_LP.

## Data Availability

All data sets used and analyzed during the current study are available: data is available at the Sequence Read Archive (PRJNA679918).

## Abbreviations

STH: Small tailed Han sheep
FP: Follicular phase
LP: Luteal phase
MM: *FecB*^*BB*^ genotype
ww: *FecB*^++^ genotype
LncRNA: Long noncoding RNA
HPT: Hypothalamic-pituitary-thyroid
HPG: Hypothalamic-pituitary-gonadal
TSH: Thyroid-stimulating hormone
FPKM: The fragments per kilobase of transcript per million reads mapped
CIDR: Controlled internal drug releasing device
