# Supplementary figures and images for: Pineal gland transcriptomic profiling reveals the differential regulation of lncRNA and mRNA related to prolificacy in STH sheep with two FecB genotypes
Source: BMC Genom Data. 2021 Feb 18;22:9. doi: 10.1186/s12863-020-00957-w (PMC7893892; doi:10.1186/s12863-020-00957-w)

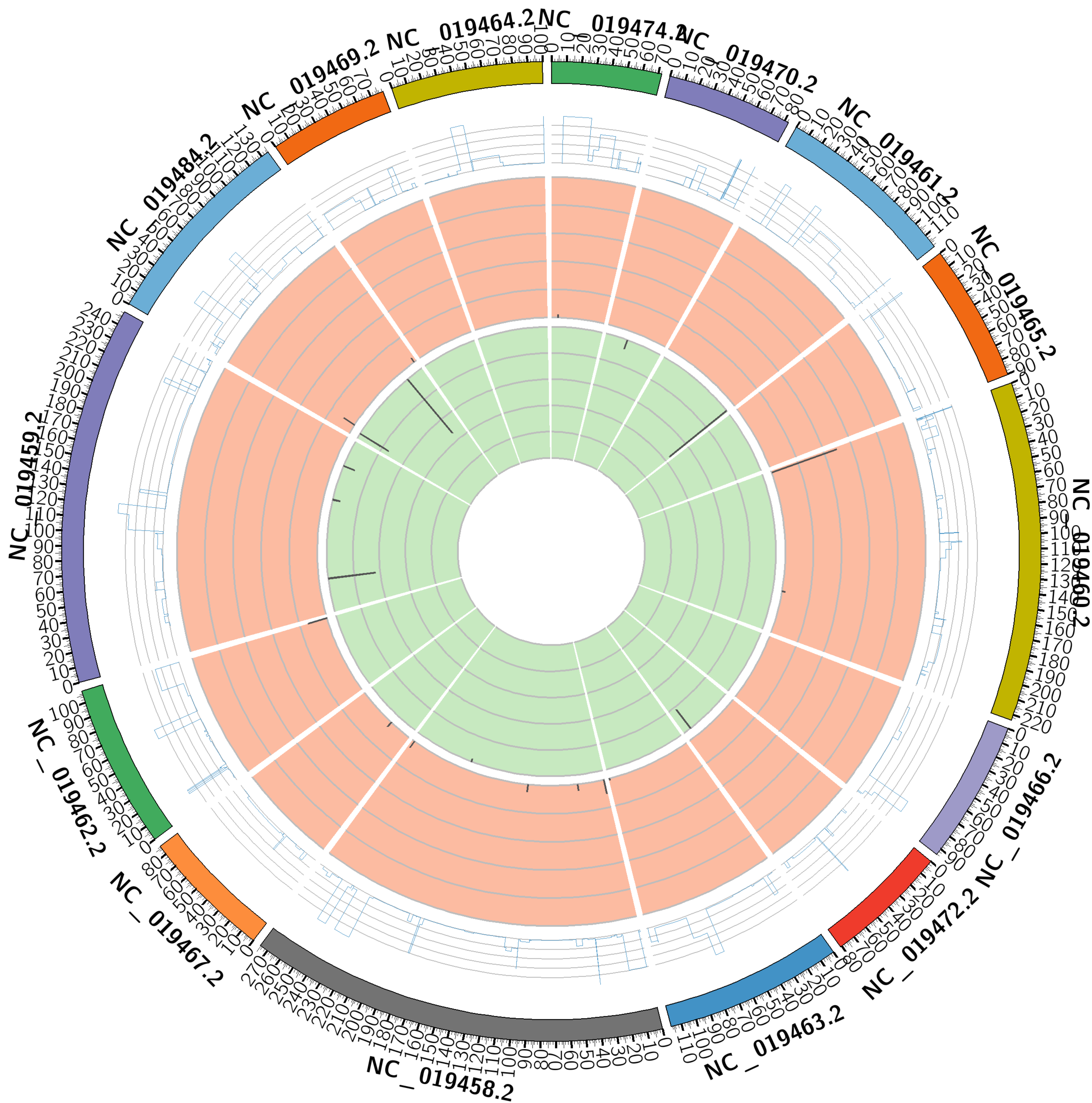

Supplement: Supplementary file 1 — Additional file 1: Figure S1. Distribution of DE lncRNAs on chromosomes in MM_FP vs MM_LP. [file 12863_2020_957_MOESM1_ESM.pdf]

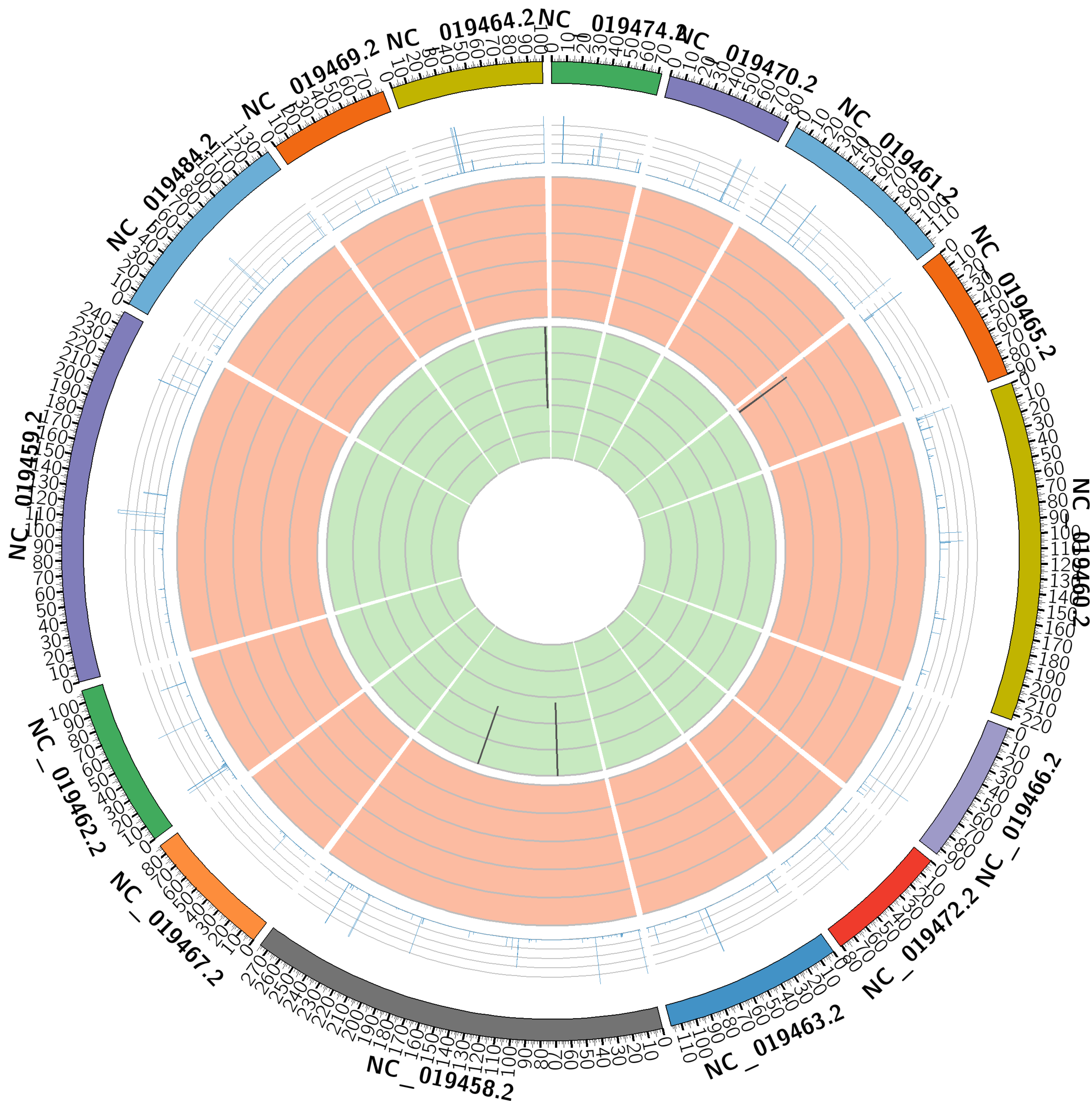

Supplement: Supplementary file 2 — Additional file 2: Figure S2. Distribution of DE lncRNAs on chromosomes in MM_FP vs ww_FP. [file 12863_2020_957_MOESM2_ESM.pdf]

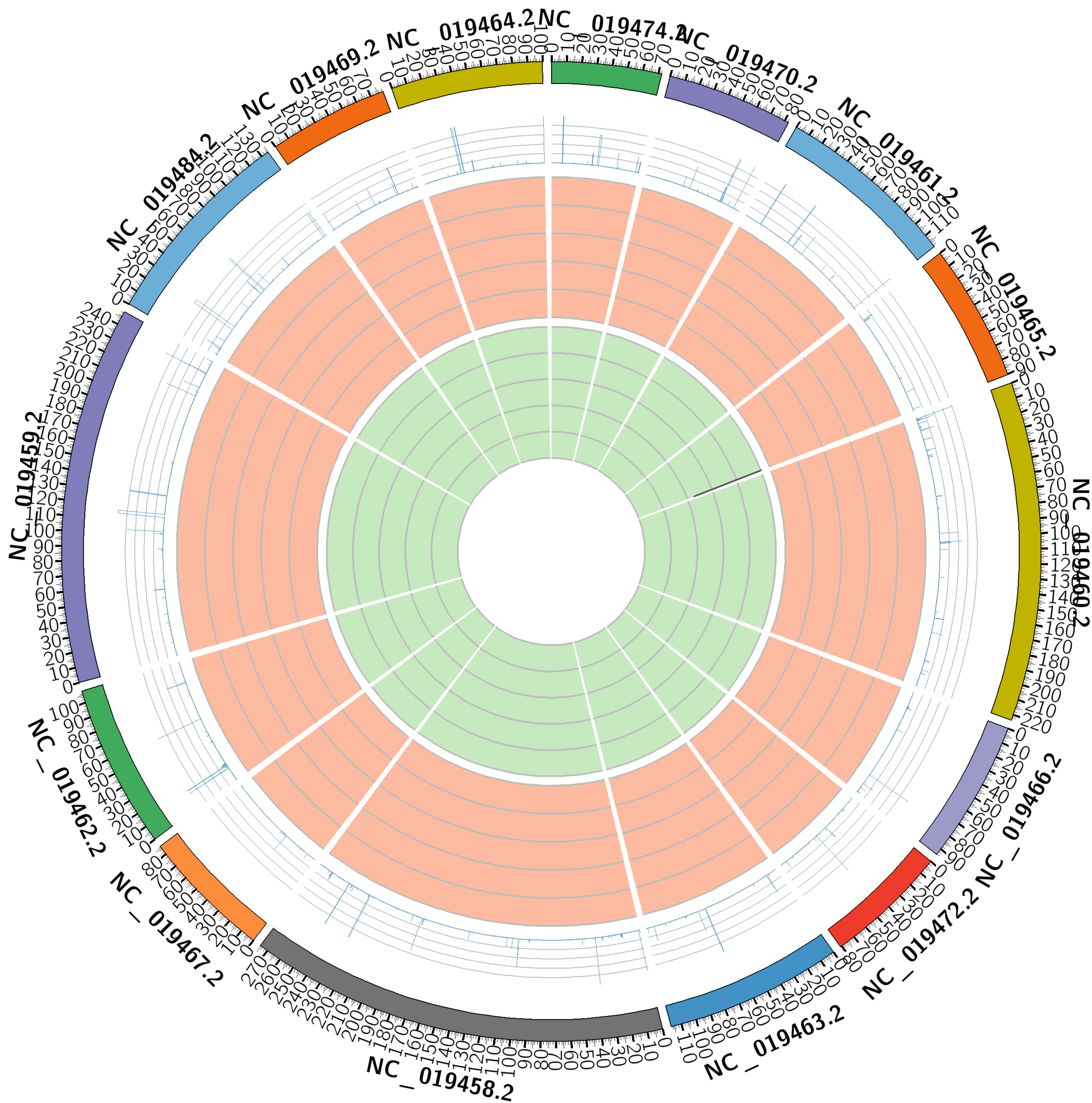

Supplement: Supplementary file 3 — Additional file 3: Figure S3. Distribution of DE lncRNAs on chromosomes in MM_LP vs ww_LP. [file 12863_2020_957_MOESM3_ESM.pdf]

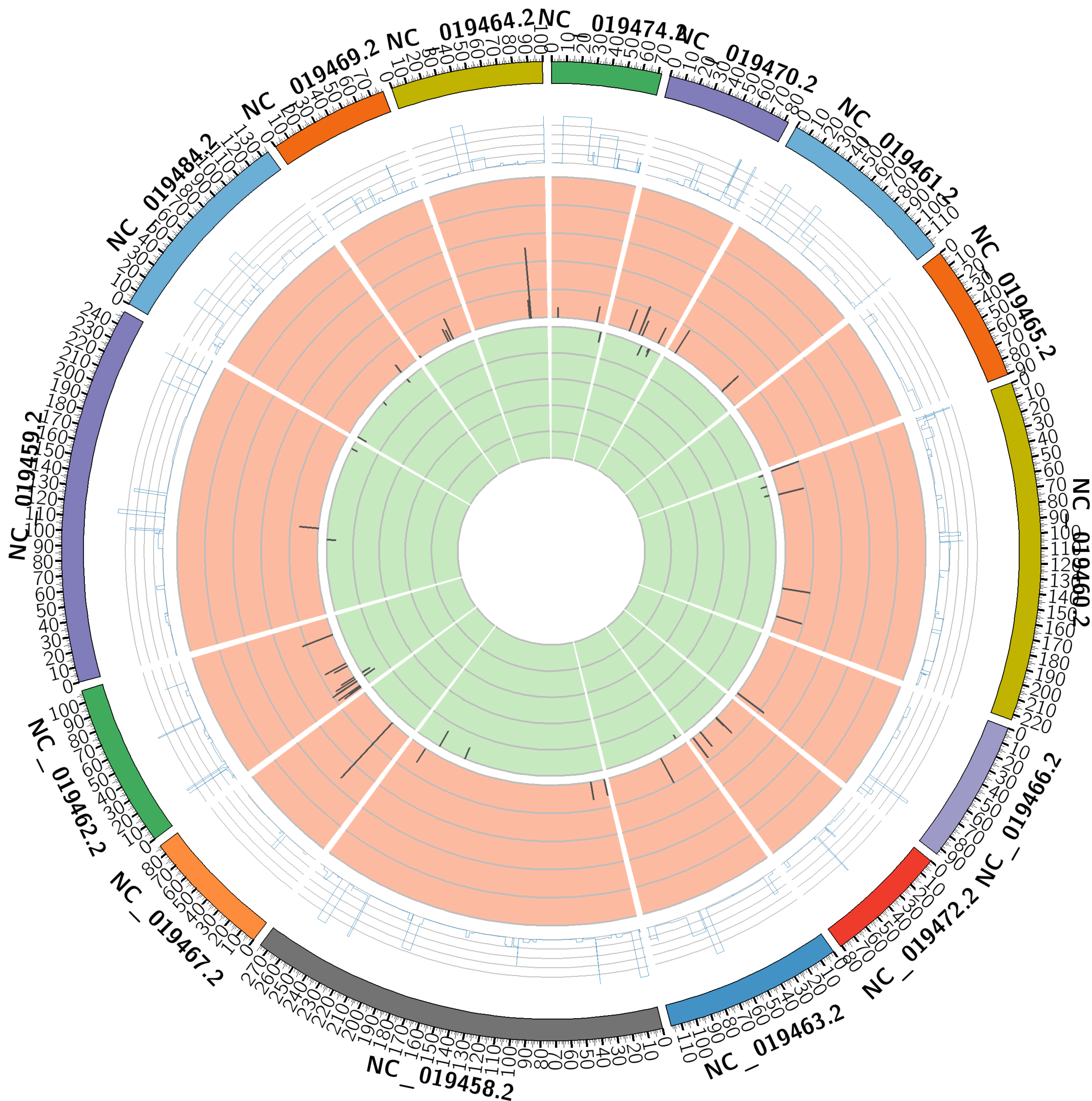

Supplement: Supplementary file 4 — Additional file 4: Figure S4. Distribution of DE lncRNAs on chromosomes in ww_FP vs ww_LP. [file 12863_2020_957_MOESM4_ESM.pdf]

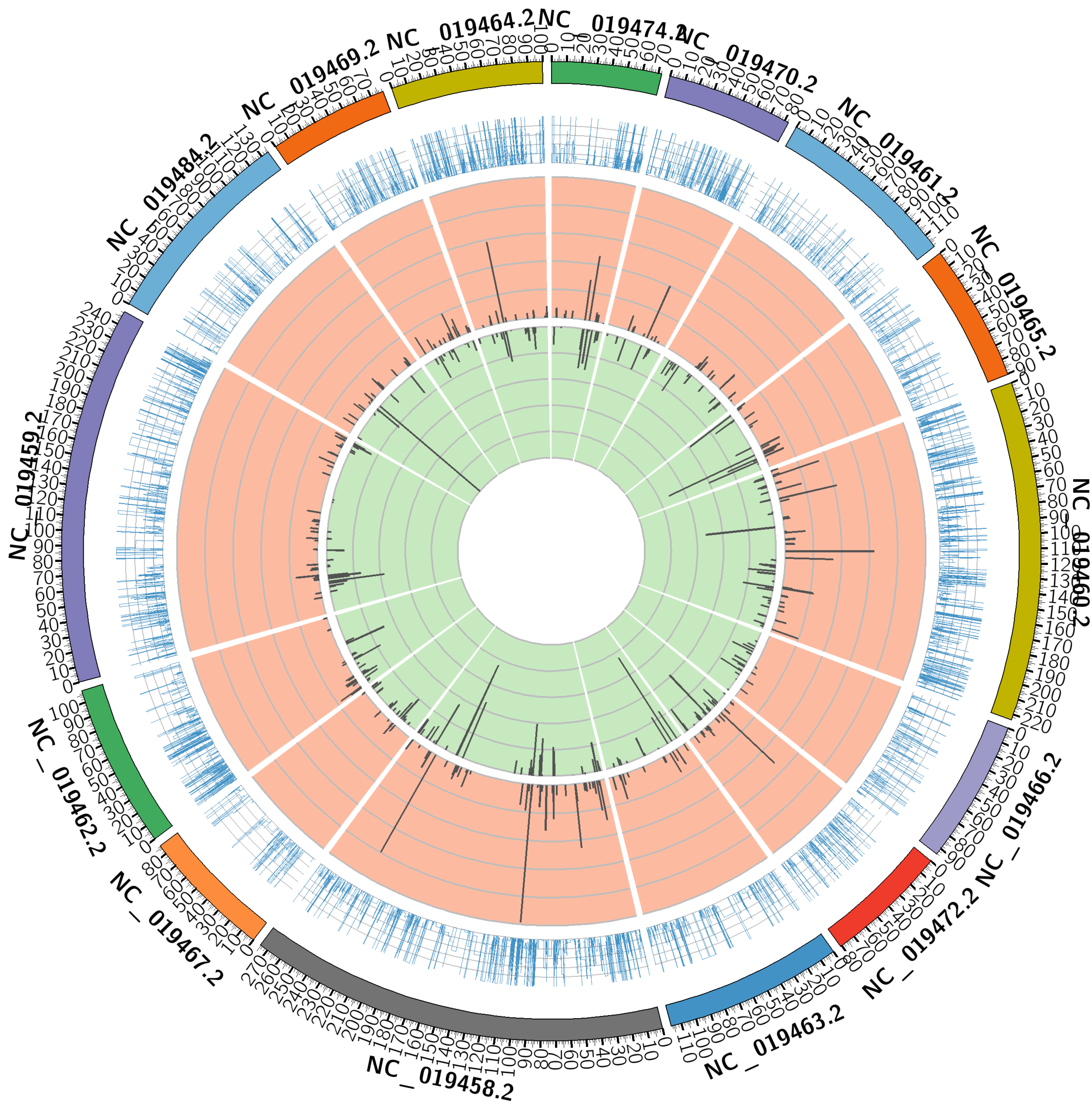

Supplement: Supplementary file 5 — Additional file 5: Figure S5. Distribution of DE mRNAs on chromosomes in MM_FP vs MM_LP. [file 12863_2020_957_MOESM5_ESM.pdf]

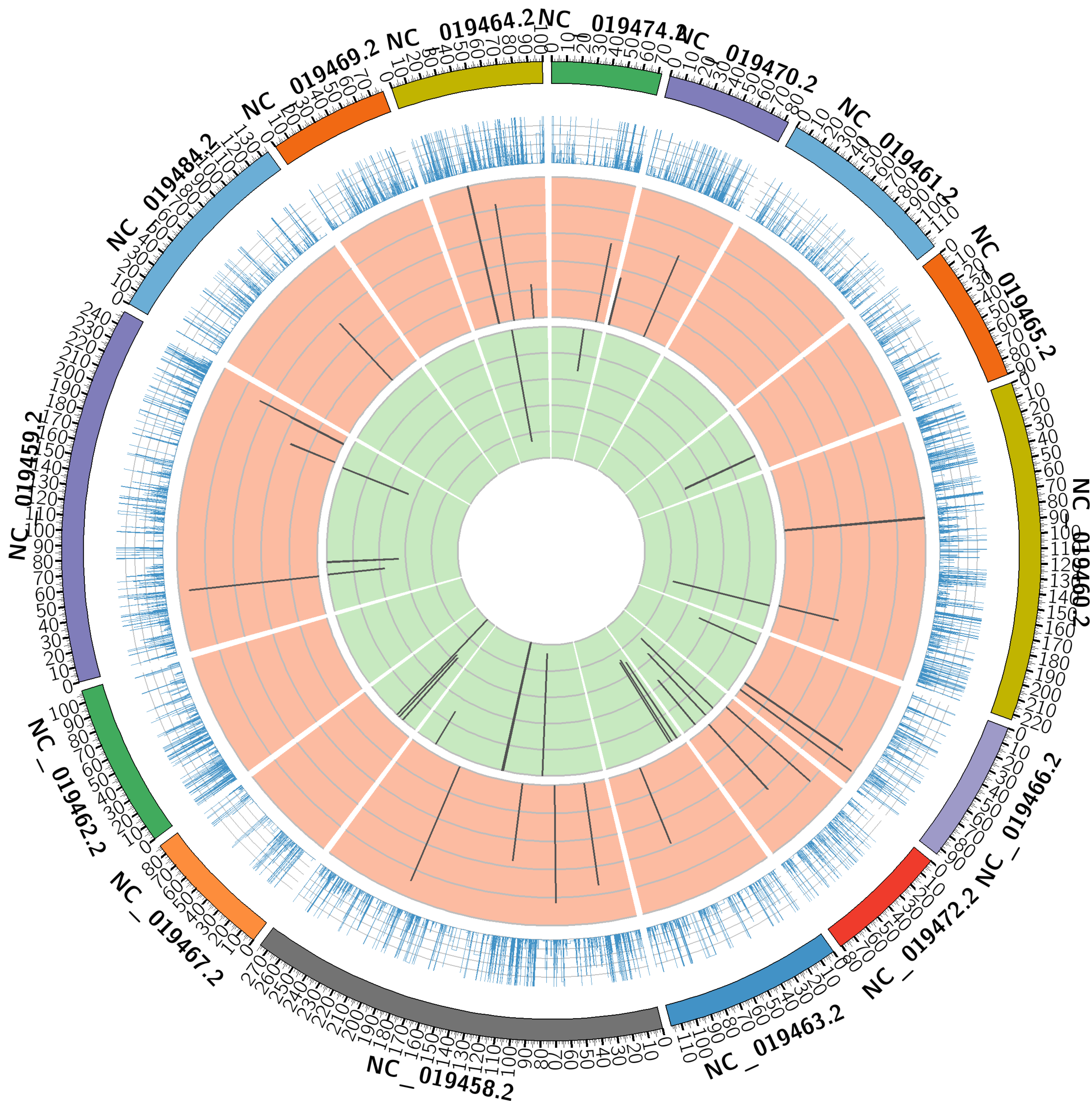

Supplement: Supplementary file 6 — Additional file 6: Figure S6. Distribution of DE mRNAs on chromosomes in MM_FP vs ww_FP. [file 12863_2020_957_MOESM6_ESM.pdf]

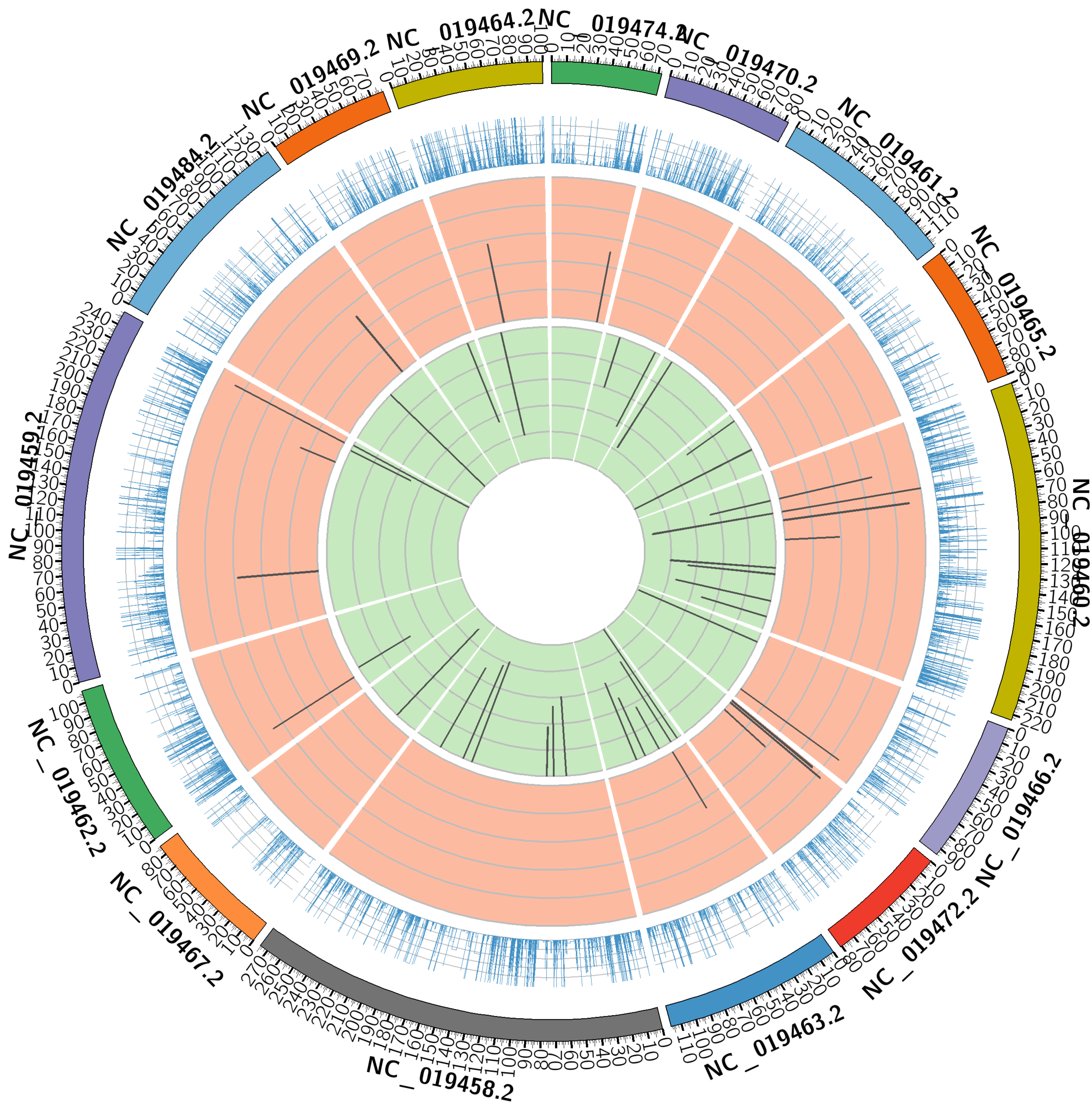

Supplement: Supplementary file 7 — Additional file 7: Figure S7. Distribution of DE mRNAs on chromosomes in MM_LP vs ww_LP. [file 12863_2020_957_MOESM7_ESM.pdf]

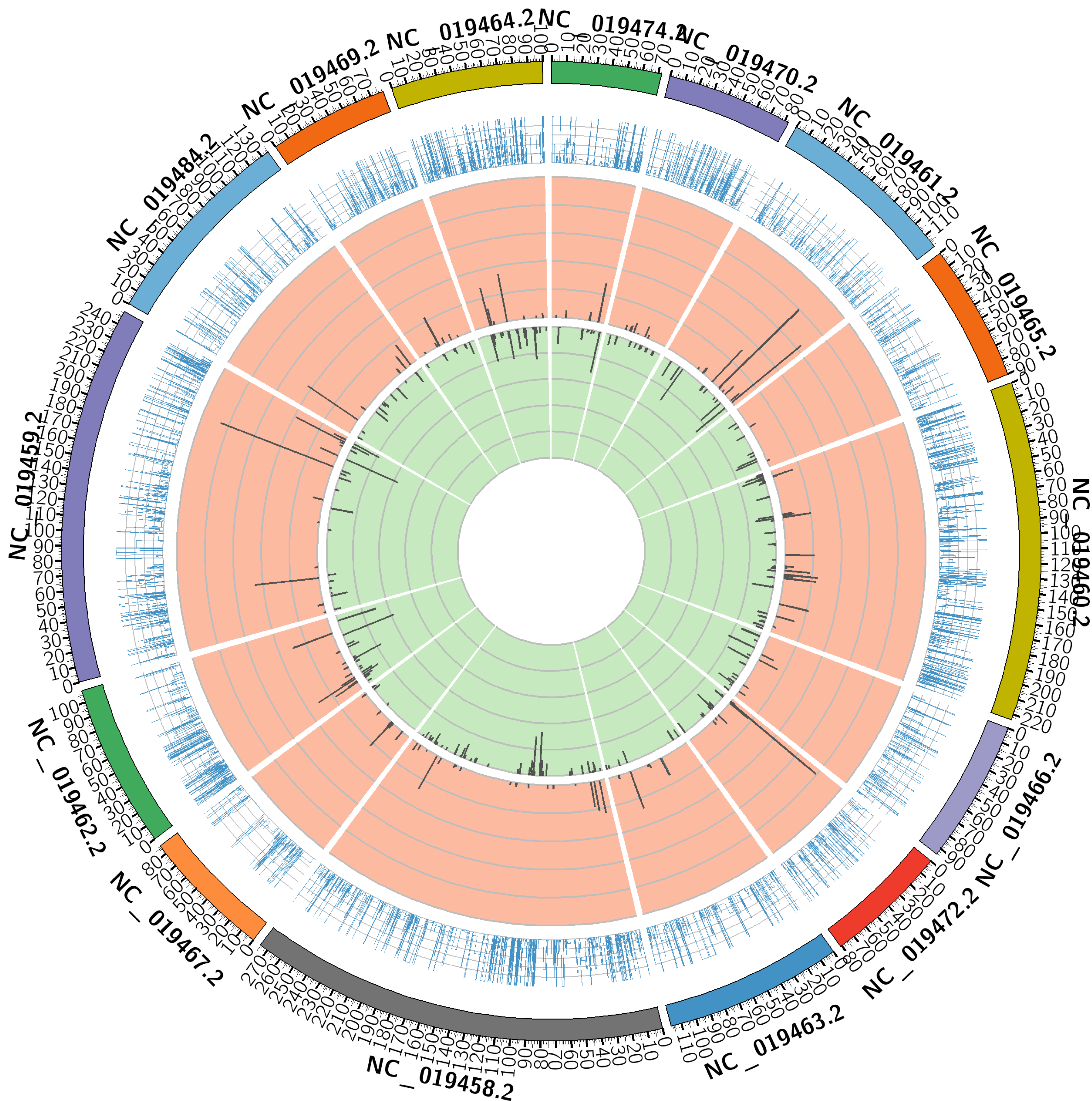

Supplement: Supplementary file 8 — Additional file 8: Figure S8. Distribution of DE mRNAs on chromosomes in ww_FP vs ww_LP. [file 12863_2020_957_MOESM8_ESM.pdf]

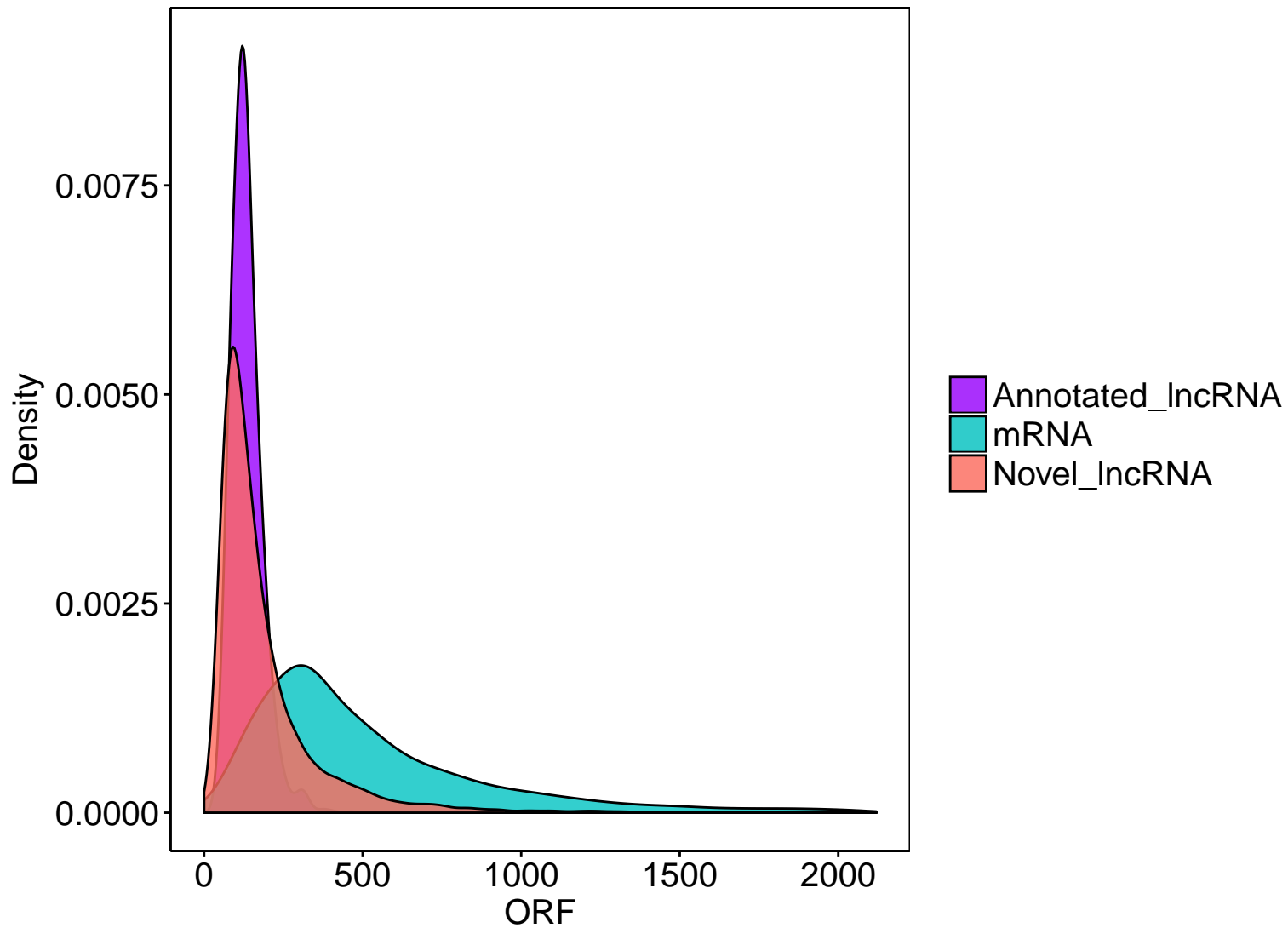

Supplement: Supplementary file 9 — Additional file 9: Figure S9. Density distribution of candidate transcripts. [file 12863_2020_957_MOESM9_ESM.pdf]
