# Supplementary material for: Pineal gland transcriptomic profiling reveals the differential regulation of lncRNA and mRNA related to prolificacy in STH sheep with two FecB genotypes
Source: BMC Genom Data. 2021 Feb 18;22:9. doi: 10.1186/s12863-020-00957-w (PMC7893892; doi:10.1186/s12863-020-00957-w)
Supplement: Supplementary file 10 — Additional file 10: Table S1. Overview of DE mRNAs closely related to reproductive signal pathways. [file 12863_2020_957_MOESM10_ESM.docx]

**Table S1 Overview of DE mRNAs closely related to reproductive signal pathways**.

|  | **Pathway term** | **Rich factor** | **Q_value** | **Gene number** | **Regulation** | **Gene _id** |
| --- | --- | --- | --- | --- | --- | --- |
| **MM_F_P vs MM_L_P** | Thyroid hormone signaling pathway | 0.067797 | 0.244565 | 8 | down | 101111097\|101118462\|101120281\|443447\|101109759\|101113846\|443519\|101104249 |
|  | MAPK signaling pathway | 0.05098 | 0.244565 | 13 | down | 101115429\|494436\|101120920\|101118462\|101117432\|443447\|101108362\|101107438\|101122427\|101113846\|101108104\|106991889\|101113917 |
|  | cGMP-PKG signaling pathway | 0.046784 | 0.468129 | 8 | down | 101110314\|101115429\|101118462\|100147789\|101108104\|101109759\|101113846\|101113297 |
| **MM_F_P vs ww_F_P** | Hippo signaling pathway | 0.019608 | 0.612308 | 3 | up | 101103028\|101120947\|443300 |
|  | Thyroid hormone synthesis | 0.028571 | 0.612308 | 2 | up | 101108200\|101103585 |
|  | Steroid biosynthesis | 0.045455 | 0.612308 | 1 | up | 101110013 |
|  | Thyroid hormone signaling pathway | 0.016949 | 0.612308 | 2 | up | 101111344\|101103585 |
|  | cGMP-PKG signaling pathway | 0.017544 | 0.689068 | 3 | down | 101102896\|101116716\|101102086 |
|  | AMPK signaling pathway | 0.015873 | 0.689068 | 2 | down | 101102896\|443270 |
| **MM_L_P vs ww_L_P** | Cell cycle | 0.014925 | 0.375098 | 2 | up | 101105870\|101105847 |
|  | MAPK signaling pathway | 0.007843 | 0.394902 | 2 | up | 101108362\|101113917 |
|  | Steroid hormone biosynthesis | 0.015152 | 0.394902 | 1 | up | 105612707 |
|  | mTOR signaling pathway | 0.015152 | 0.394902 | 1 | up | 101119321 |
|  | Hippo signaling pathway | 0.019608 | 0.712031 | 3 | down | 101120947\|554322\|101110782 |
|  | GnRH signaling pathway | 0.022989 | 0.712031 | 2 | down | 443453\|101106371 |
|  | Oxytocin signaling pathway | 0.019108 | 0.712031 | 3 | down | 443453\|101113784\|101106371 |
|  | Circadian entrainment | 0.020833 | 0.712031 | 2 | down | 443453\|101106371 |
| **ww_F_P vs ww_L_P** | Hippo signaling pathway | 0.03268 | 0.837175 | 5 | up | 101101932\|100294600\|443417\|100913160\|101102148 |
|  | Phototransduction | 0.068966 | 0.757058 | 2 | down | 101109549\|101115216 |
|  | Circadian rhythm | 0.0625 | 0.757058 | 2 | down | 101104483\|100169937 |
|  | Hippo signaling pathway | 0.03268 | 0.757058 | 5 | down | 101116012\|101117599\|100294600\|100913160\|101116743 |
